# The new DPJO: New forms of publishing with the same commitment to science

**DOI:** 10.1590/2177-6709.25.6.007-008.edt

**Published:** 2020

**Authors:** Flavia Artese, Laurindo Zanco Furquim

**Affiliations:** 1 Universidade do Estado do Rio de Janeiro, Departamento de Odontologia Preventiva e Comunitária (Rio de Janeiro/RJ, Brazil).; 2 Universidade Estadual de Maringá, Departamento de Odontologia (Maringá/PR, Brazil).

For those who were born and raised before the digital age, it is evident that nowadays information spreads itself extremely fast and effectively. In this universe of available data, generally within reach of the palm of our hands, it is sometimes difficult to separate false from truth. Communication today can be false, distorted and biased and still be dispersed in great speed, without frontiers or limits, gaining the same strength as what should be trusted.

In this scenario, scientific journals have gained force as a north, since their main objective is to disseminate information from the scientific world that has been analyzed and certified through peer reviewing. In addition, publications guarantee authorship and origin of the work, as well as serve as public archives for future searches.^1^ In other words, well-structured scientific journals are essential educational tools that can ultimately improve the treatment of patients.^2^

The editorial process of scientific journals dates back to 1665 when the first issue of the Philosophical Transactions of the Royal Society was published and included nine articles, a dedication, a list of books and other correspondences.^1^ Although the purpose of publications have been the same since then, the process of publishing a journal has improved over time, especially due to peer reviewing and search tools.

On the other hand, the academic pressure for scientific publication seems to blur the real purpose of the publication itself. This is understandable and legitimate regarding the publication of articles resulting from research, which are generally financed by development agencies. In addition, the number and quality of publications by a researcher or professor classifies him within the academic environment. This demand led to a great proliferation of journals of the same area, which ended up also being classified through the most diverse indexes, such as the impact factor.

Therefore, in this universe of multiple options, the editorial team of a journal is concerned with its ranking, in order to be chosen by authors with submissions of relevant articles. The DPJO was founded 25 years ago as a local journal, published in Portuguese, by a private publisher. Since then it has been published uninterruptedly every two months and has always tried to improve to maintain its focus: the dissemination of information within the area of Orthodontics.

Along these 25 years, editors and reviewers have worked voluntarily to transform a local journal into a periodical with international reach. The DPJO is currently an open-access journal, published in English, and indexed in the main scientific databases. In the SCImago Journal Ranking the DPJO is the tenth journal in the area of orthodontics and the third open access journal in this area in the world.

This growth of the DPJO has attracted a significant number of submissions from all over the world in the past 5 years. Due to the limited space for publication and the demand for approved articles, the final publication time is longer than desired. In order to expedite our publications, the DPJO decided to make a series of changes. Starting in the first edition of 2021, the journal will no longer exist in print and will be published in a continuous flow model. That is, the articles will be made available in the databases before the completion of a number. This more modern model provides greater flexibility in the authors’ publication and immediate access to the journal’s readers. The sections by guest authors will also be reduced in number, allowing the publication of more original articles, and editorials will be published only in special situations. In addition, to celebrate its 25 years of existence, the DPJO will start 2021 with a new format, completely digital and adapted for tablets and smartphones.

This current year was surprised by instant changes and we participated in the establishment of an even more digital and possibly faster world. In this way, the DPJO’s new formatting and editing process are adapted to these new times, but respecting the good old practices. With these changes, the journal reaffirms its commitment to science, to authors and readers, hoping for an even higher ranking and relevance for the journal. However, we continue with our main objective: to guarantee qualified information, in a democratic way, to those who have a commitment to orthodontics based on scientific evidence.

Long live the DPJO.

## References

[1] Rallison SP (2015). What are journals for. Ann R Coll Surg Engl.

[2] Marusic M, Marusic A (2009). The purpose of scientific journals Small is
Important. J Teh Univ Heart Ctr.

